# Does gender affect intraocular lens power calculations? A comparative analysis of formula accuracy

**DOI:** 10.25122/jml-2025-0091

**Published:** 2025-06

**Authors:** Ionela-Iasmina Yasar, Servet Yasar, Leila Al Barri, Diana-Maria Darabus, Alina Iasmina Dumitriu, Mihnea Munteanu, Horia Tudor Stanca

**Affiliations:** 1 Department IX–Surgery I, Victor Babes University of Medicine and Pharmacy Timisoara, Romania; 2 Prof. Dr. Munteanu Ophthalmologic Center, Timisoara, Romania; 3 Department 12–Ophthalmology, Otorhinolaryngology, Carol Davila University of Medicine and Pharmacy, Bucharest, Romania

**Keywords:** intraocular lens power calculation formulas, Kane, Barrett Universal II, Hill-Radial Basis Function, Ladas Super Formula, spherical equivalent, AI, Artificial Intelligence, IOL, Intraocular Lens, AL, Axial Length, ACD, Anterior Chamber Depth, SS-OCT, Swept-Source Optical Coherence Tomography, BU II, Barrett Universal II, Hill-RBF, Hill-Radial Basis Function, LSF, Ladas Super Formula, SE, Spherical Equivalent, EDOF, Extended Depth of Focus, WTW, White-to-White, CCT, Central Corneal Thickness, K, Keratometry, LT, Lens Thickness

## Abstract

Intraocular lens (IOL) power calculation is a crucial aspect of modern cataract surgery, directly affecting postoperative refractive outcomes. Due to anatomical and physiological differences between male and female eyes, neglecting gender-specific variations may lead to systematic refractive errors in IOL power selection. This research aimed to determine the necessity of incorporating gender as a variable in future IOL calculation algorithms to improve postoperative precision. This retrospective observational study analyzed data from 210 eyes that met predefined inclusion criteria. Biometric measurements for assessing intraocular lens power were obtained using the ARGOS optical biometer. The refractive power of the intraocular lenses was calculated using several established formulas, and these values were systematically compared to evaluate their predictive accuracy. A parametric statistical approach was employed, using repeated measures ANOVA to assess differences among multiple calculations. The homogeneity of covariances was examined to validate the design of the repeated measures. Pairwise comparisons of the outcomes from different formulas were performed using the Bonferroni correction to identify statistically significant differences. Additionally, paired-sample *t*-tests were conducted to compare the spherical equivalent values recorded during the initial and final follow-up examinations. Significant differences were observed between genders regarding descriptive features such as age, axial length, keratometry, and white-to-white measurements. Although notable biometric differences exist between genders, they do not seem to affect the discrepancies in intraocular lens power calculations using the tested formulas. However, intra-group differences in intraocular power values suggest that the choice of formula may impact predicted intraocular power differently for men and women.

## INTRODUCTION

Accurate intraocular lens (IOL) power calculation remains a cornerstone of contemporary cataract surgery, exerting a direct impact on postoperative refractive outcomes. Although established biometric parameters, such as axial length (AL), keratometry (K), and anterior chamber depth (ACD), are routinely employed in IOL power prediction, the potential influence of biological sex has not been extensively examined [1-3]. Given the well-documented anatomical and physiological distinctions between male and female eyes, disregarding sex-specific biometric differences may contribute to systematic refractive inaccuracies during lens selection [2,4].

The present study investigated the extent to which gender-related biometric disparities affect the accuracy of intraocular lens (IOL) power predictions. Specifically, it compared the outputs of four commonly utilized calculation formulas: the Kane formula, Barrett Universal II (BU II), Hill-Radial Basis Function (Hill-RBF), and Ladas Super Formula (LSF), across male and female patient cohorts.

Prior ophthalmologic research has consistently highlighted sex-based differences in ocular anatomy [4-6]. Women, on average, tend to have shorter axial lengths, shallower anterior chambers, and steeper corneal curvatures than men [7,8]. These anatomical differences raise concerns regarding the applicability of current IOL power calculation methods, which primarily rely on biometric variables without incorporating sex as an explicit factor. For instance, findings by Zhang *et al*. indicate a higher incidence of postoperative hyperopic errors in women, attributed mainly to their shorter axial lengths and deviations in the estimation of effective lens position (ELP) [9]. Furthermore, minor differences in corneal power between sexes may lead to measurable variations in spherical equivalent (SE), reinforcing the rationale for incorporating biological sex into IOL prediction models [10,11].

Despite the growing body of evidence highlighting these sex-based ocular differences, most contemporary IOL formulas do not explicitly consider gender as an independent variable. This study undertakes a systematic evaluation of the Kane, Barrett Universal II, Hill-RBF, and Ladas Super Formula models to determine whether sex-specific biometric variation has a significant impact on refractive outcomes. By assessing predictive accuracy across both male and female populations, this research aimed to ascertain the necessity of integrating gender as a variable in future IOL calculation algorithms to enhance postoperative precision.

The implications of these findings are substantial for the advancement of precision cataract surgery. Incorporating gender-specific calibration or adjusting existing formulas to account for sex-based anatomical differences could enhance refractive accuracy. As innovations in artificial intelligence and machine learning continue to refine IOL power prediction, addressing gender-based variability may represent a pivotal step in minimizing residual refractive error and optimizing visual outcomes.

## MATERIAL AND METHODS

### Study design and participants

This retrospective observational study aimed to evaluate the refractive outcomes following the successful implantation of IOLs. The research was conducted at the Department of Ophthalmology, Victor Babeș University of Medicine and Pharmacy in Timisoara, Romania, from July 2021 to June 2024. Before the surgery, all participants underwent a comprehensive ophthalmologic examination to establish their baseline refractive status and assess overall ocular health. The study included individuals who received IOL implants for refractive correction or cataract treatment. To ensure consistency in measurements, a single examiner conducted all postoperative refractive evaluations in one session, taking three consecutive measurements to obtain an average for analysis. Biometric assessments to determine IOL power were performed using the ARGOS optical biometer. Refractive power was calculated using four established formulas: Kane, BU II, Hill-RBF, and the LSF. These methods were systematically compared, a testament to the thoroughness of our methodology, to evaluate their relative predictive accuracy. Postoperative assessments were scheduled at two intervals: an early follow-up between one and three months after surgery and a later evaluation between three and twelve months post-surgery. At each visit, the SE was calculated. Patients who did not attend follow-up appointments within the specified timeframes were excluded from the final data analysis. Descriptive statistical analyses of the two most used lens brands and their subtypes classified according to their focal points were also performed.

### Study population

This study analyzed a total of 210 eyes that met the predetermined inclusion criteria. The sample consisted of 175 participants, including 101 women and 74 men.

### Exclusion criteria

Participants were excluded if they had a documented history of ocular trauma, ocular surgery, AL measurement of less than 21 mm or greater than 26 mm, corneal or vitreous opacities, diagnosis of dry eye syndrome, retinal pathology, diagnosis of glaucoma or nystagmus, and did not attend follow-up evaluations.

### Surgical procedure

In accordance with the methodology described in BU II, IOLs were selected to achieve a postoperative SE as close to zero as possible. The surgical procedure involved creating a primary corneal incision at an angle of 110 degrees with a width of 2.2 mm. Additionally, two auxiliary side port incisions measuring 1.2 mm in width were made at 30 and 160 degrees, respectively. The study incorporated various types of IOLs, including monofocal lenses, extended depth of focus (EDOF) lenses, and trifocal lenses. The most frequently used IOLs, renowned for their quality and innovation, were produced by leading manufacturers such as Alcon (Alcon Laboratories, Fort Worth), Zeiss (Carl Zeiss Meditec), and Johnson & Johnson (New Brunswick).

### ARGOS

The ARGOS swept-source optical coherence tomography (SS-OCT) system operates at a central wavelength of 1060 nm with a spectral bandwidth of 20 nm, achieving an A-scan acquisition rate of 3,000 scans per second. This allows for the generation of high-resolution, two-dimensional OCT images. Keratometric data are obtained using a 2.2 mm diameter ring comprising 16 light-emitting diodes (LEDs). The reflected image from this LED ring, combined with the OCT signal, enables precise measurement of corneal curvature, applying a corneal refractive index of 1.3375. The system applies a corneal refractive index of 1.3375 for these measurements. The ARGOS SS-OCT system extracts corneal diameter directly from the OCT image and uses it as a reference parameter for estimating the white-to-white (WTW) distance. This is done through imaging protocols established by ALCON Laboratories (Fort Worth), which are designed to ensure accurate measurements. The device also employs OCT technology to determine anterior chamber depth (ACD), lens thickness (LT), and axial length (AL), each calculated using specific refractive indices for the ocular media: 1.376 for the cornea, 1.336 for both the aqueous and vitreous humor, and 1.410 for the crystalline lens.

### Groups

*Group 1:* The Kane formula used in this study was accessed from the latest version of the website.

*Group 2:* The Hill-RBF method was employed for IOL calculation, using the latest online version at the time of analysis.

*Group 3:* The BU II formula was utilized directly from the most recent version integrated into the ARGOS device on the day of surgery.

*Group 4:* The LSF formula was applied using the most current version accessible via its official website [13].

### Statistical analysis

Statistical analysis was performed using IBM SPSS Statistics, version 25 (IBM Corp., Armonk, NY, USA). Descriptive data were summarized using frequencies, percentages, and means with standard deviations, medians, and minimum and maximum values. The normality of data distribution was assessed via the Kolmogorov–Smirnov test. The data conformed to a normal distribution, meaning that it is symmetric and follows a bell-shaped curve, allowing for the use of parametric statistical techniques that assume a normal distribution. Accordingly, repeated measures analysis of variance (ANOVA) was employed to evaluate within-subject differences. A sphericity test was applied to assess the assumption of sphericity (i.e., the equality of variances of the differences between repeated measures). In instances where this assumption was satisfied, standard *P* values were reported, demonstrating our commitment to statistical rigor; otherwise, the Greenhouse–Geisser correction was utilized to adjust the degrees of freedom. Furthermore, meticulous post hoc analyses were conducted using the Bonferroni correction to determine the pairwise comparisons responsible for statistically significant effects. Paired-sample *t*-tests were also performed to compare IOL power and SE values. Statistical significance was defined as a *P* value less than 0.05.

## RESULTS

In this study, a total of 210 eyes were analyzed. It is important to note that not all individuals contributed data from both eyes; however, 35 participants provided bilateral eye data. Consequently, the final analysis included 128 eyes from female participants and 82 eyes from male participants. The mean age of the female participants was 64.18 ± 10.54 years, while the mean age for male participants was 62.18 ± 12.06 years. There was no significant difference in age between the two groups. However, we found significant differences between genders for the descriptive features related to age, AL, K1, and WTW measurements (*P* values of 0.01, 0.02, and 0.01, respectively). No significant differences were observed in K2, ACD, and LT (Table 1).

**Table 1:** Descriptive statistics

		Min	Max	Mean ± SD	*P*
**Age**	F	39	86	64.18 ± 10.54	0.21
	M	40	91	62.18± 12.06	
**AL**	F	21	25.77	23.12 ± 88.13	0.01
	M	21.73	24.99	23.52 ± .70	
**K1**	F	40.31	48.33	43.81 ± 1.54	0.02
	M	39.99	46.93	43.18 ± 1.24	
**K2**	F	40.50	48.89	44.45 ± 1.64	0.07
	M	40.87	47.39	43.89 ± 1.26	
**ACD**	F	2.52	4.11	3.16 ± .33	0.06
	M	2.52	4.11	3.29 ± .32	
**LT**	F	3.45	5.49	4.52 ± .36	0.61
	M	3.81	5.53	4.54 ± .38	
**WTW**	F	10.80	12.90	11.87 ± .37	0.01
	M	11.25	12.93	12.04 ± .35	

AL, Axial Length; K, Keratometry; ACD, Anterior Chamber Depth; Lt, Lens Thickness; WTW, White-to-White.

The most frequently used IOL brands in this study were ALCON and ZEISS. Among the ALCON lenses, the different subtypes included monofocal, extended depth of focus (EDOF), and trifocal lenses, with monofocal lenses being the most frequently utilized. Similarly, ZEISS lenses included both monofocal and trifocal subtypes, with monofocal lenses being more commonly used than trifocal ones (Table 2).

**Table 2:** Descriptive statistics of the two most frequently used lens brands and their subtypes classified by focal points

	*n*	%
ALCON*	129	61.1
Monofocal**	58	44.9
EDOF**	23	17.8
Trifocal**	48	37,3
ZEISS*	44	20,8
Monofocal**	28	36,6
Multifocal**	16	63,4

* The frequency distribution of all brands utilized in the study. ** The frequency distribution within the ALCON/ZEISS brand.

**Figure 1:**
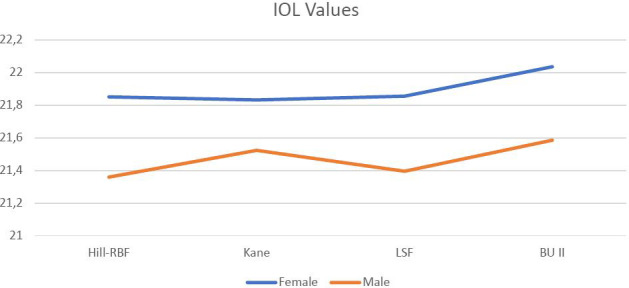
IOL power values

IOL power values calculated using Kane, Hill-RBF, BU II, and LSFs were compared for female and male participants. No statistically significant differences were observed between genders for any of the formulas applied (Table 3, Figure 1).

**Table 3:** Comparison of formulas within themselves in terms of gender

		Mean± SD	*P*
**Hill-RBF**	M	21.85 ± 2.26	0.09
	F	21.35 ± 1.80	
**Kane**	M	21.83 ± 2.31	0.31
	F	21.52 ± 1.90	
**LSF**	M	21.85 ± 2.27	0.12
	F	21.39 ± 1.82	
**BU II**	M	22.03 ± 2.27	0.13
	F	21.58 ± 1.83	

The IOL power values calculated using the Kane, Hill-RBF, BU II, and LSF methods were compared between female and male groups. A significant difference in the values was observed in both groups (*P* < 0.01). Further subgroup analyses revealed that, in females, the BU II formula yielded higher values compared to the Kane, Hill-RBF, and LSF methods. In males, the Kane and BU II values were comparable. Overall, the Kane and BU II values were significantly higher in men (Table 4).

**Table 4:** IOL power values calculated using formulas were compared in female and male groups

		Mean ± SD	df	F	*P*
**Female**	Hill-RBF	21.80 ± 0.200	3	25.52	<0.01 (BU II > Hill-RBF, Kane, LSF)
	Kane	21.83 ± 0.205			
	LSF	21.85 ± 0.202			
	BU II	22.03 ± 0.201			
**Male**	Hill-RBF	21.35 ± 1.80	2.74	19.99	<0.01 (Kane, BU II > Hill RBF, LSF)
	Kane	21.52 ± 1.90			
	LSF	21.39 ± 1.82			
	BU II	21.58 ± 1.83			

Potential correlations between the formulas were analyzed for both groups using Pearson correlation analysis. The results indicated a statistically significant positive correlation between the data obtained from the formulas. This correlation was found to be strong in both groups (Table 5).

**Table 5:** Correlation analysis between BU II and LSFs

Female		Hill-RBF	Kane	LSF	BU II
**Hill-RBF**	Pearson Corr.	1	.992(**)	.991(**)	.992(**)
	Sig. (2-tailed)		.000	.000	.000
**Kane**	Pearson Corr.	.992(**)	1	.990(**)	.992(**)
	Sig. (2-tailed)	.000		.000	.000
**LSF**	Pearson Corr.	.991(**)	.990(**)	1	.990(**)
	Sig. (2-tailed)	.000	.000		.000
**BU II**	Pearson Corr.	.992(**)	.992(**)	.990(**)	1
	Sig. (2-tailed)	.000	.000	.000	
**Male**		**Hill-RBF**	**Kane**	**LSF**	**BU II**
**Hill-RBF**	Pearson Corr.	1	.990(**)	.989(**)	.989(**)
	Sig. (2-tailed)		.000	.000	.000
**Kane**	Pearson Corr.	.990(**)	1	.985(**)	.986(**)
	Sig. (2-tailed)	.000		.000	.000
**LSF**	Pearson Corr.	.989(**)	.985(**)	1	.982(**)
	Sig. (2-tailed)	.000	.000		.000
**BU II**	Pearson Corr.	.989(**)	.986(**)	.982(**)	1
	Sig. (2-tailed)	.000	.000	.000	

**Figure 2:**
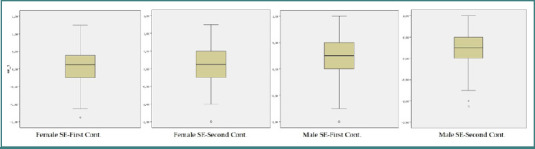
SE values in boxplot graphic

SE values determined in both groups' first and second controls were compared. SE values were significantly higher in men than in women (*P* = 0.038 and 0.034, respectively) (Table 6, Figure 2).

**Table 6:** Comparison of SE values calculated during the first and second postoperative follow-ups

SE First Control	Mean ± SD	Std. Error	t	df	*P*
Female	0.07 ± 0.50	0.044	-2,01	208	0.038
Male	0.21 ± 0.2	0.047			
**SE Second Control**	**Mean±SD**	**Std. Error**	**T**	**df**	***P***
Female	0.09 ± 0.41	0.045	-2,08	208	0.034
Male	0.23 ± 0.45	0.05			

## DISCUSSION

The present study aimed to investigate the influence of gender on IOL power calculation by comparing four commonly used formulas: Kane, BU II, Hill-RBF, and LSF. Our findings demonstrate that although specific biometric parameters, such as AL, flat keratometry (K1), and WTW measurements, differed significantly between genders, the calculated IOL power values were broadly consistent across both male and female groups. However, significant variations were noted within gender groups when comparing formulas, with Kane and BU II yielding higher values in men [12-14]. In comparison, BU II values were higher than those of the other formulas in women. Additionally, SE values were significantly greater in men. These results align with some prior research while also highlighting potential inconsistencies in the impact of gender on IOL power calculations [15].

Our findings regarding biometric differences between genders are consistent with previous studies reporting that men typically exhibit longer ALs and larger WTW values than women. At the same time, ACD and LT are similar across genders. For instance, Cooke *et al*. found that AL was significantly greater in men, which could influence IOL power predictions, given the known association between AL and refractive outcomes [15]. Similarly, Narvaez *et al*. reported significant differences in corneal diameter and AL between male and female patients, suggesting that biometric variations may need to be accounted for in IOL power calculations [16]. However, in our study, despite these biometric discrepancies, the IOL power values derived from Kane, BU II, Hill-RBF, and LSF formulas did not differ significantly between genders, suggesting that these modern formulas may already account for such variations in their computational algorithms [17,18].

Interestingly, while gender did not directly influence IOL power calculations, formula-specific differences emerged within each gender. In women, BU II yielded higher values than Kane, Hill-RBF, and LSF. In men, Kane and BU II produced comparable values, and Hill-RBF was similar to LSF. The higher BU II values in women may reflect the formula's design, incorporating a broader range of biometric parameters, potentially amplifying subtle anatomical differences [19]. In contrast, the similarity between Kane and BU II values in men may indicate that these formulas function similarly in cases with longer AL, as supported by previous reports emphasizing the accuracy of Kane and BU II in longer eyes [20].

Furthermore, our analysis of spherical equivalent outcomes revealed that men exhibited significantly higher SE values than women at both postoperative evaluations. This finding suggests a potential gender-dependent variation in postoperative refractive outcomes, which may be influenced by biometric factors such as AL and corneal curvature. Previous studies, including those by Melles *et al*., have indicated that men may experience a greater tendency for myopic shifts postoperatively, potentially contributing to the observed SE differences [21]. SE differences align with our findings and underscore the necessity of further research to determine whether gender-specific adjustments in refractive targets may be warranted in some instances.

Lastly, our correlation analysis revealed strong positive correlations between the IOL power values obtained from different formulas, reinforcing their general consistency in predicting refractive outcomes. This result supports the robustness of modern IOL power calculation formulas, as previously documented by Kane *et al*., who found that contemporary formulas exhibit high inter-formula agreement despite variations in algorithmic complexity [22].

## CONCLUSION

In conclusion, while significant biometric differences exist between genders, they do not appear to translate into discrepancies in IOL power calculations using the Kane, BU II, Hill-RBF, or LSF formulas. However, intra-group differences in IOL power values suggest that formula selection may influence predicted IOL power differently in men and women. Additionally, the observed gender differences in spherical equivalent values emphasize the need for further research to optimize refractive outcomes. Future studies incorporating larger sample sizes and diverse populations may clarify whether gender-specific refinements in IOL power calculation strategies are warranted to enhance postoperative visual outcomes.

## Data Availability

The data supporting the findings of this study are available from the corresponding author upon reasonable request.

## Personal thanks

We want to acknowledge Victor Babes University of Medicine and Pharmacy Timisoara for their support in partially covering the costs of publication for this research paper.
